# Molecular and serological detection of *Leptospira* spp. in wildlife from Paraíba, Brazil

**DOI:** 10.3389/fvets.2026.1904553

**Published:** 2026-07-23

**Authors:** Ividy Bison, Audisio A. Costa Filho, Glenison F. Dias, José Rômulo S. Santos, Jeann L. Araújo, Maria Luana Cristiny R. Silva, Sérgio Santos De Azevedo, Roberta N. Parentoni, Lucas Rannier Ribeiro Antonino Carvalho, Arthur Willian De Lima Brasil

**Affiliations:** 1 Postgraduate Program in Animal Science, Federal University of Paraíba, Paraíba, Brazil; 2 Brazilian Institute of the Environment and Renewable Natural Resources, João Pessoa, Brazil; 3 Academic Unit of Veterinary Medicine, Federal University of Campina Grande, Paraíba, Brazil; 4 Department of Physiology and Pharmacology, Karolinska Institutet, Stockholm, Sweden

**Keywords:** Brazil, epidemiological surveillance, *Leptospira* spp., microscopic agglutination test, One Health, wild mammals, zoonosis

## Abstract

Leptospirosis is a widespread zoonotic disease caused by pathogenic *Leptospira* spp., affecting humans, domestic animals, and wildlife. Wild mammals are recognized as potential maintenance hosts for pathogenic *Leptospira* spp., and some species may act as asymptomatic reservoirs, representing a potential public health risk, especially in areas with increasing human-wildlife interaction. This cross-sectional study investigated the occurrence of pathogenic *Leptospira* spp. in wild mammals from Paraíba, Brazil, sampled between June 2022 and June 2023 using serological and molecular approaches. Blood samples from 60 animals were analyzed by the microscopic agglutination test (MAT), while tissue samples from 29 animals were evaluated by polymerase chain reaction (PCR) targeting the *lipL32* gene, a marker specific for pathogenic *Leptospira* spp. Seroreactivity was detected in 9/60 animals (15.0%), with reactions against the serogroups Pomona, Hebdomadis, Grippotyphosa, Shermani, Patoc, and Pyrogenes. Pathogenic *Leptospira* DNA was detected in 6/29 animals (20.7%), with positive PCR results obtained from multiple tissues, including the kidneys, bladder, liver, brain, and reproductive organs. These findings demonstrate exposure to and infection by pathogenic *Leptospira* spp. in wild mammals from Paraíba and reinforce the importance of wildlife surveillance for a better understanding of the epidemiology of leptospirosis.

## 1 Introduction

Many wildlife species serve as reservoirs for zoonotic pathogens. Increasing contact between wildlife, humans, and domestic animals may facilitate pathogen transmission, contributing to the emergence of new disease patterns and changes in disease occurrence and pathogenicity (1). Therefore, the growing interface between human populations and wildlife represents a public health concern and should be continuously monitored (2).

From a One Health perspective, surveillance of zoonotic pathogens in wildlife is essential, as pathogen circulation at the wildlife–domestic animal–human interface may influence animal, environmental, and public health. Monitoring *Leptospira* spp. in wild populations can improve our understanding of pathogen circulation and support strategies aimed at reducing the potential risk of spillover to domestic animals and humans.

Among zoonotic diseases, leptospirosis is an important re-emerging and neglected bacterial disease that affects domestic animals, wildlife, and humans. Pathogenic *Leptospira* spp., the causative agents of leptospirosis, are widely distributed in wildlife and have been reported in reptiles, birds, and especially wild mammals (3, 4).

Wildlife infections with *Leptospira* spp. are frequently subclinical, with many infected animals showing no evident clinical signs (5). This characteristic may hinder the detection and epidemiological surveillance of leptospirosis in free-ranging populations.

The transmission of leptospirosis occurs through direct or indirect contact with the urine of infected animals, through which the pathogen can penetrate both damaged and intact skin. Other routes of transmission have also been identified, such as the ingestion of contaminated food or water (6).

Despite the recognized importance of wildlife in the epidemiology of leptospirosis, information on the occurrence and circulation of *Leptospira* spp. in free-ranging animals from northeastern Brazil, particularly in the state of Paraíba, remains limited. Furthermore, combining serological methods, such as the microscopic agglutination test (MAT), with molecular techniques, such as PCR, provides complementary evidence by identifying previous exposure through serology and the presence of *Leptospira* DNA through molecular detection, thereby strengthening epidemiological surveillance.

Given the recognized importance of wildlife surveillance for leptospirosis and the limited information available for free-ranging animals in northeastern Brazil, this study aimed to investigate the occurrence of *Leptospira* spp. infection in rescued wildlife from the state of Paraíba using serological (MAT) and molecular (PCR) methods, and to describe the distribution of MAT-reactive serogroups.

## 2 Materials and methods

### 2.1 Study area

**Figure 1:**
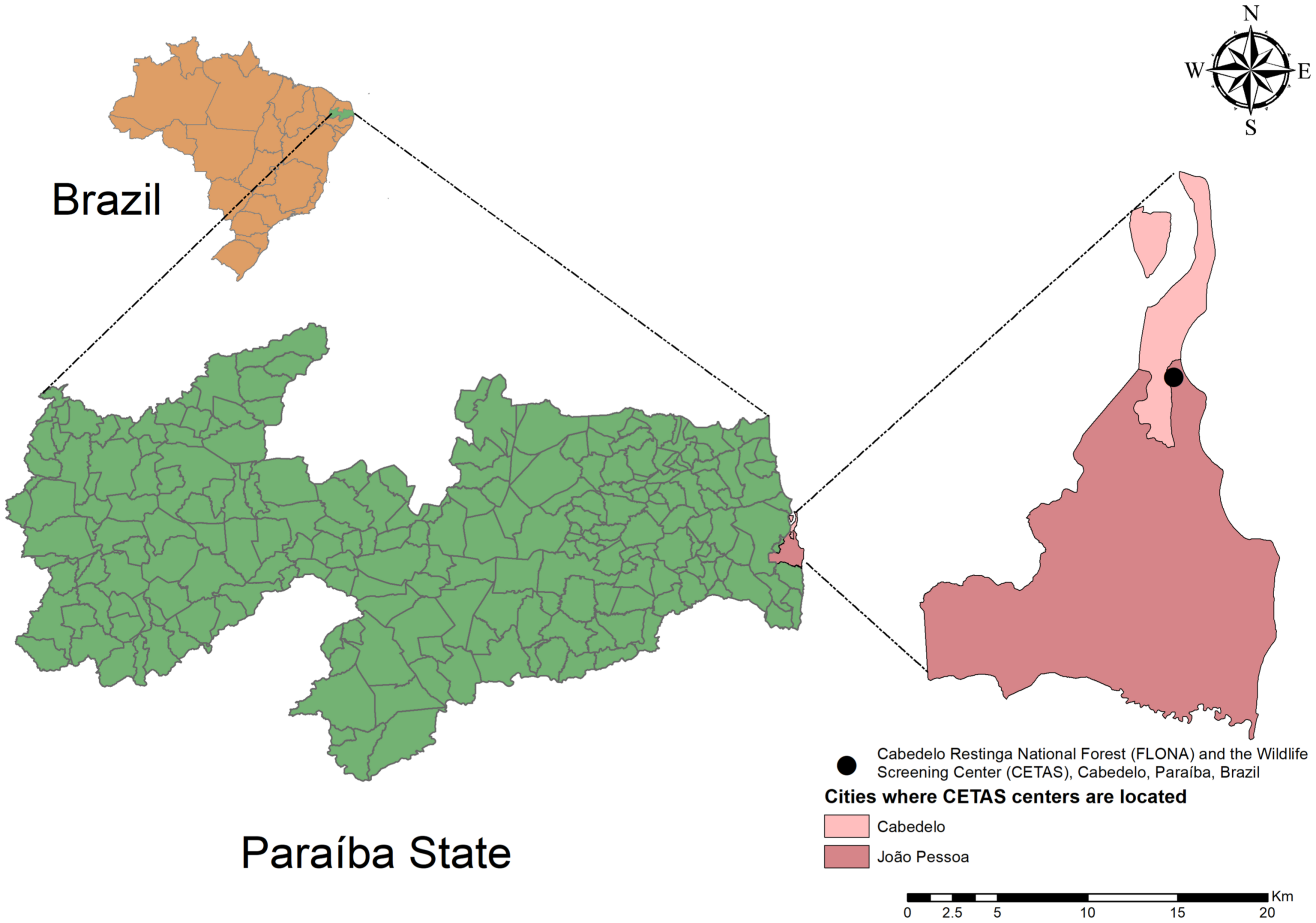
Geographic location of the Wildlife Screening Center (CETAS) in the state of Paraíba, northeastern Brazil, where wild animals included in this study were maintained and sampled. The map highlights the municipalities of Paraíba state and the location of CETAS within the Cabedelo Restinga National Forest (FLONA), in the municipality of Cabedelo, near João Pessoa.

The animals included in this study were obtained through rescue and seizure operations conducted by the Environmental Military Police of the State of Paraíba and the Brazilian Institute of Environment and Renewable Natural Resources (IBAMA) across different municipalities of Paraíba state. These animals originated from a variety of ecological settings, including urban, peri-urban, and rural areas, as well as natural and fragmented habitats of the Atlantic Forest biome, reflecting different degrees of anthropogenic influence and human–wildlife interaction. After rescue or seizure, the animals were transported to the Wildlife Screening Center (CETAS) in Cabedelo, Paraíba, located within a 103-hectare fragment of Atlantic Forest (ICMBio), where they were maintained under veterinary care until rehabilitation, reintroduction, or transfer to licensed zoological institutions. Sample collection was performed during their stay at this facility (Figure 1).

### 2.2 Animal and biological sample collection

The sample size was determined by convenience sampling, based on the number of wild animals admitted to CETAS during the study period and the availability of biological samples suitable for serological and molecular analyses. Due to the opportunistic nature of wildlife rescue and rehabilitation activities, a formal sample size calculation was not feasible.

A total of 60 blood samples were collected from wild animals belonging to 12 species, including capuchin monkeys (*Sapajus* spp.) (*n* = 24), marmosets (*Callithrix jacchus*) (*n* = 9), southern tamanduas (*Tamandua tetradactyla*) (*n* = 7), opossums (*Didelphis albiventris*) (*n* = 5), crab-eating foxes (*Cerdocyon thous*) (*n* = 3), raccoons (*Procyon cancrivorus*) (*n* = 3), coatis (*Nasua nasua*) (*n* = 2), porcupines (*Coendou prehensilis*) (*n* = 2), jaguarundis (*Herpailurus yagouaroundi*) (*n* = 2), six-banded armadillos (*Euphractus sexcinctus*) (*n* = 1), nine-banded armadillos (*Dasypus novemcinctus*) (*n* = 1), and capybaras (*Hydrochoerus hydrochaeris*) (*n* = 1).

Serum samples were subjected to the microscopic agglutination test (MAT), a serological assay used for the detection of antibodies against *Leptospira* spp., the etiological agent of leptospirosis. Venipuncture sites varied according to species and included cephalic, lateral and medial caudal, femoral, and jugular veins. Approximately 2 mL of blood was collected per animal, depending on body size. After collection, samples were centrifuged, stored at the Clinical Analysis Laboratory of CETAS, and subsequently sent for serological analysis.

Tissue samples were obtained by necropsy from 29 animals, including marmosets (*Callithrix jacchus*) (*n* = 9), opossums (*Didelphis albiventris*) (*n* = 5), porcupines (*Coendou prehensilis*) (*n* = 3), southern tamanduas (*Tamandua tetradactyla*) (*n* = 3), jaguarundis (*Herpailurus yagouaroundi*) (*n* = 3), capuchin monkeys (*Sapajus* spp.) (*n* = 1), agoutis (*Dasyprocta leporina*) (*n* = 1), crab-eating foxes (*Cerdocyon thous*) (*n* = 1), six-banded armadillos (*Euphractus sexcinctus*) (*n* = 1), capybaras (*Hydrochoerus hydrochaeris*) (*n* = 1), and nine-banded armadillos (*Dasypus novemcinctus*) (*n* = 1).

Both blood and tissue samples were collected from some animals, including southern tamanduas (*n* = 2), opossums (*n* = 2), capybaras (*n* = 1), and nine-banded armadillos (*n* = 1), as these animals died during the study period after admission to CETAS.

### 2.3 Serological diagnosis

The detection of anti-*Leptospira* spp. antibodies was performed using the microscopic agglutination test (MAT). A panel of 19 serogroups was used as antigens, namely Australis, Autumnalis, Ballum, Bataviae, Canicola, Celledoni, Cynopteri, Djasiman, Grippotyphosa, Hebdomadis, Icterohaemorrhagiae, Javanica, Panama, Pomona, Pyrogenes, Semaranga, Sejroe, Shermani, and Tarassovi. Serological diagnosis was carried out in two stages: screening and determination of antibody titers. During the screening stage, all serum samples were tested against the 19 antigens using a cutoff value of 1:100. Reactive sera were subsequently retested to determine the final antibody titers through serial dilutions in a geometric progression up to a maximum dilution of 1:3,200. The final titer was defined as the reciprocal of the highest dilution showing at least 50% agglutination of leptospires (7). All analyses were performed at the Laboratório multiusuário of the Department of Morphology at the Federal University of Paraíba, located in João Pessoa, Paraíba, Brazil.

### 2.4 Molecular identification

Molecular detection of *Leptospira* spp. was performed on tissue samples collected during necropsy from animals that died during the study period. Tissue fragments were frozen immediately after collection and transported to the Laboratório de Doenças Transmissíveis at the Federal University of Campina Grande for analysis. Tissue samples were collected from the liver, kidneys, bladder, lungs, heart, central nervous system, uterus, and gonads. Bacterial DNA was extracted from all tissue samples using the DNeasy^®^ Blood and Tissue Kit (Qiagen, Hilden, Germany), the manufacturer’s instructions. Molecular detection of *Leptospira* spp. was performed by conventional polymerase chain reaction (PCR) targeting the *lipL32* gene, according to the protocol described by Stoddard et al. (7). The *lipL32* gene is present exclusively in pathogenic *Leptospira* species. The primers used were LipL32-45F (5′-AAGCATTACCGCTGGTG-3′) and LipL32-286R (5′-GAACTCCCATTTCAGCGATT-3′), which amplify a 242-bp fragment. PCR reactions were carried out in a final volume of 50 μL containing 5 μL of extracted DNA, 27.8 μL of UltraPure DNase/RNase-Free Distilled Water, 10 μL of reaction buffer, 1.0 μL of dNTPs, 3.0 μL of MgCl₂, 1.5 μL of each primer (LipL32-45F and LipL32-286R), and 0.2 μL of Taq DNA polymerase. Amplification was performed under the following cycling conditions: initial denaturation at 94 °C for 5 min, followed by 35 cycles of denaturation at 94 °C for 30 s, annealing at 53 °C for 30 s, and extension at 72 °C for 60 s, with a final extension at 72 °C for 5 min. *Leptospira interrogans* serogroup Pomona, serovar Kennewicki, was used as the positive control, whereas UltraPure DNase/RNase-Free Distilled Water served as the negative control. PCR products were considered positive when a band of the expected size (242 bp) was visualized under ultraviolet light following electrophoresis on a 2% agarose gel stained with Blue Green Loading Dye I and compared with a molecular weight marker.

### 2.5 Ethical approval

The present study was approved by the Chico Mendes Institute for Biodiversity Conservation (ICMBio) under authorization issued by the Biodiversity Authorization and Information System (SISBIO), registration number 83589–2. Furthermore, following the Ethical Principles for Animal Experimentation established by the National Council for the Control of Animal Experimentation (CONCEA), the study was approved by the Animal Use Ethics Committee (CEUA) of the Federal University of Paraíba, protocol number 8156061022.

### 2.6 Statistical analysis

Descriptive statistical analyses included the calculation of absolute and relative frequencies of seropositive and PCR-positive animals according to species and sample type, as well as 95% confidence intervals (95% CI) for seroprevalence and PCR positivity rates using the exact binomial method. Due to the limited sample size and heterogeneous distribution of species, no inferential statistical analyses were performed.

## 3 Results

Of the 60 animals included in the study, all were tested by serology (MAT). A subset of 29 animals, which died during the study period, were additionally evaluated by molecular analysis (PCR) of tissue samples. Therefore, some animals were tested by both methods, while others were evaluated only by MAT.

An overall seroprevalence of 15% (9/60, 95% CI: 7.1–26.6) was observed among the evaluated animals. The reactive animal species, serogroups, and antibody titers are presented in Table 1. Antibody titers ranged from 100 to 3,200, with the following serogroups detected: Pomona (3/9), Hebdomadis (2/9), Grippotyphosa (1/9), Shermani (1/9), Patoc (1/9), and Pyrogenes (1/9).

**Table 1:** Reactive *Leptospira* serogroups and corresponding MAT antibody titers detected in seropositive wild animals from Paraíba State, northeastern Brazil.

Species	Animal ID	Serogroups					
		Pomona	Grippotyphosa	Patoc	Shermani	Hebdomadis	Pyrogenes
Capuchin monkey (*Sapajus* spp.)	S1	1:1,600					
Capuchin monkey (*Sapajus* spp.)	S2		1:1,600				
Capuchin monkey (*Sapajus* spp.)	S3					1:1,600	
Capuchin monkey (*Sapajus* spp.)	S4	1:1,600					
Capuchin monkey (*Sapajus* spp.)	S5			1:400			
Opossum (*Didelphis albiventris*)	DA5	1:3,200					
Southern tamandua (*Tamandua tetradactyla*)	TT1				1:3,200		
Crab-eating fox (*Cerdocyon thous*)	CT1					1:3,200	
Marmoset (*Callithrix jacchus*)	CJ1						1:100
Total positive animals by serogroup		3	1	1	1	2	1

The presumptive infecting serogroup for each animal was defined according to the highest MAT antibody titers.

Primates of the genus *Sapajus* spp. exhibited the highest number of positive individuals in the microscopic agglutination test (MAT), accounting for 55.5% (5/9) of all reactive animals. In addition to primates, one individual of *Didelphis albiventris* tested positive for the Pomona serogroup, exhibiting a high antibody titer (1:3,200). High antibody titers (>1:400) were observed in eight (8/9) of the positive animals. The animals evaluated in this study did not present clinical signs suggestive of leptospirosis.

**Figure 2:**
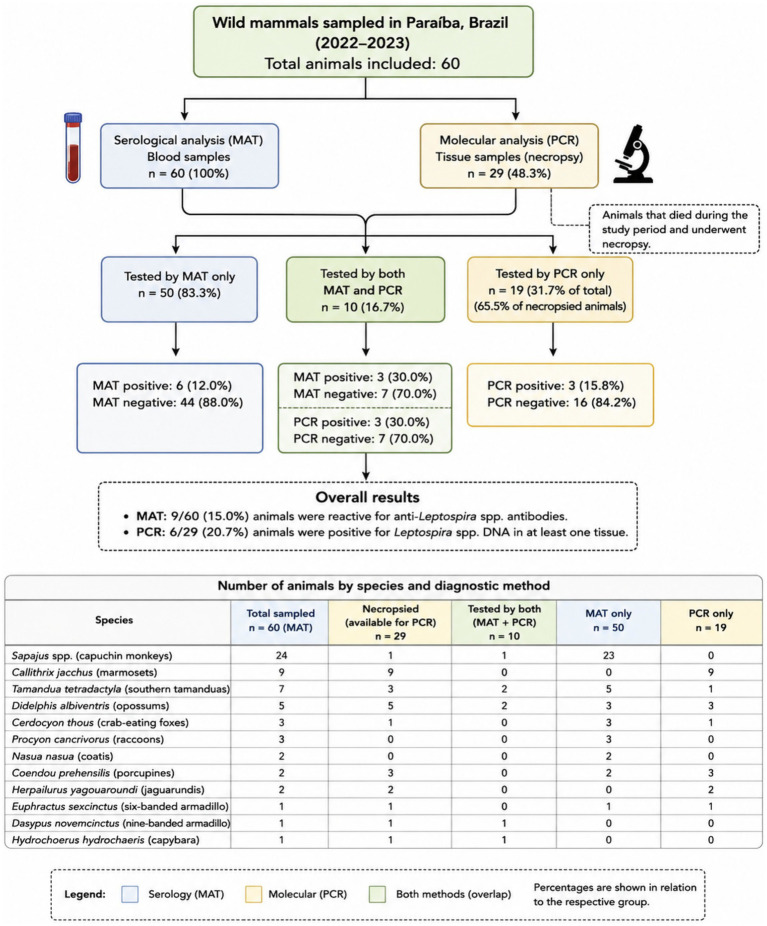
Distribution of the 60 wild mammals included in the study according to diagnostic method. All animals were tested by MAT, while PCR was performed on tissue samples from 29 necropsied animals. The figure shows the overlap between animals tested by both methods, those evaluated exclusively by MAT or PCR, and the corresponding positive and negative results for each diagnostic approach.

In the molecular analysis of tissues performed by polymerase chain reaction (PCR), 6 (20.7%; 95% CI: 8.0–39.7) of the 29 wild mammals showed DNA amplification for *Leptospira* spp. in at least one of the analyzed organs. PCR positivity for the *lipL32* gene was detected in three marmosets (3/9), two jaguarundis (2/2), and one capybara (1/1) (Figure 2).

PCR results are presented according to the 29 animals that underwent necropsy, with tissue samples analyzed from multiple organs per individual. The denominators presented in Table 2 refer to the number of positive animals among those evaluated for each species.

**Table 2:** Distribution of tissues positive for *Leptospira* DNA by *lipL32* PCR among wild mammals from Paraíba State, Brazil, between 2022 and 2023.

Organ	Number of animals positive for *lipL32* by tissue		
	Marmoset (*n* = 3) (*C. jacchus*)	Jaguarundi (*n* = 2) (*H. yagouaroundi*)	Capybara (*n* = 1) (*H. hydrochaeris*)
Kidneys	1	2	1
Bladder	1	2	–
Liver	1	1	–
Lung	1	1	–
Heart	1	1	1
Brain	–	2	–
Uterus	1	–	–
Gonad	2	1	

Among the animals evaluated by both MAT and PCR, concordant and discordant results were observed. Animals were classified as MAT positive/PCR positive, MAT positive/PCR negative, and MAT negative/PCR positive, demonstrating differences between serological evidence of exposure and molecular detection of pathogenic *Leptospira* DNA.

## 4 Discussion

*Sapajus* spp. have previously been reported to be seroreactive to *Leptospira* spp. in different regions of Brazil, both in captive and free-ranging populations, with antibodies detected predominantly against the Patoc, Icterohaemorrhagiae, Canicola, and Autumnalis serogroups (8, 9). The present study reports the first seroreactive Sapajus spp. from the state of Paraíba, with antibodies detected mainly against the Hebdomadis, Grippotyphosa, Patoc, and particularly the Pomona serogroups, thereby contributing to the epidemiological understanding of leptospirosis in this region.

Similarly, seroreactivity to the Pomona serogroup was observed in *Didelphis albiventris*, with a high antibody titer suggesting previous exposure to *Leptospira* spp. The Pomona serogroup has been associated with domestic and wild mammals, including swine, in different epidemiological contexts (10, 11). However, because ecological and habitat data for the sampled animals were not available in the present study, the specific sources of exposure could not be determined.

Didelphids are highly adaptable mammals and are frequently observed in peri-domestic and urban environments in search of food and shelter (12). In the state of Paraíba, *Didelphis albiventris* has been reported in squares, parks, and wooded areas with frequent human activity (13), which may increase opportunities for environmental exposure to *Leptospira* spp.

Antibodies against the Grippotyphosa and Hebdomadis serovars have already been identified in free-ranging wild animals (11). In the present study, three animals, two capuchin monkeys (*Sapajus* spp.) (2/24) and one crab-eating fox (*Cerdocyon thous*) (1/3), were seroreactive to these serovars. The presence of antibodies against *Leptospira* spp. serovars has been previously reported in crab-eating foxes in southern Brazil (14, 15). *Cerdocyon thous* is considered a highly adaptable species and has been reported in a wide variety of habitats, including areas influenced by human activity (16). Additionally, these animals may share water sources with domestic species, such as cattle, which are also known to be reactive to the Hebdomadis serovar (17). However, limited information is available on the role of the crab-eating fox in the epidemiological cycle of leptospirosis, particularly in the Northeast region of Brazil.

One lesser anteater (*Tamandua tetradactyla*) was identified with antibodies against the Shermani serogroup in the present study. In Paraíba, Sousa et al. (5) previously reported an outbreak of leptospirosis in lesser anteaters at the Parque Zoobotânico Arruda Câmara. However, the epidemiological conditions associated with captive environments may differ substantially from those observed in free-ranging populations. Lesser anteaters may come into contact with rodents or share water sources with reservoir animals in both captive and free-ranging environments, potentially increasing exposure to *Leptospira* spp.

The Shermani serogroup belongs to *Leptospira santarosai*, a pathogenic species. In addition to the lesser anteater, other free-ranging mammals have been identified with antibodies against this agent, including rodents and marsupials (18). This serogroup has also been reported in domestic mammals in Brazil, such as cattle, horses, and swine (19).

High antibody titers (>1:400) were detected in eight of the nine seropositive animals (8/9). In endemic regions, MAT titers ≥1:100 are commonly used as evidence of exposure to *Leptospira* spp. in domestic animals; however, serological results alone do not allow discrimination between recent and past infections, nor do they confirm active infection or bacterial shedding (20). None of the animals evaluated in this study presented clinical signs suggestive of leptospirosis at the time of sampling. This finding may reflect prior exposure or subclinical contact with the pathogen, although the lack of clinical manifestations should be interpreted with caution given the limitations of serology. PCR testing was performed only in a subset of animals, and therefore no definitive conclusions regarding renal colonization or environmental shedding can be drawn.

With respect to the molecular analysis of tissues, PCR positivity across multiple organs suggests systemic dissemination of *Leptospira* spp. in the infected animals evaluated in this study. In particular, CNS-associated PCR positivity was observed in *Herpailurus yagouaroundi*, with detection of leptospiral DNA in brain tissue (2/2 individuals) as well as in additional organs, indicating widespread tissue distribution in this species.

In contrast, *Didelphis albiventris* was not among the PCR-positive species in the present investigation, and therefore no evidence of central nervous system infection was observed in this species in our dataset. Reports of CNS involvement in *D. albiventris* have been previously described in the literature (21), although such manifestations remain uncommon and are more frequently reported in human cases of leptospirosis (22). Experimental studies in animal models have also suggested that detection of *Leptospira* DNA in the brain may occur during early or severe systemic infection (23); however, these interpretations cannot be directly extrapolated to the present field-based observations.

In jaguarundis (*Herpailurus yagouaroundi*), PCR amplification of *Leptospira* spp. DNA was detected in multiple organs, indicating systemic infection in the individuals evaluated in this study. Evidence of exposure to *Leptospira* spp. in wild and captive felids has been reported in the literature; however, such studies often rely on serological methods and involve different species and ecological contexts, limiting direct comparison with the molecular findings presented here.

Felids are carnivorous predators and may be exposed to *Leptospira* spp. through ecological interactions with infected prey, particularly small mammals such as rodents, which are recognized reservoirs of the pathogen. In addition, environmental exposure in habitats subject to flooding or high moisture may contribute to indirect contact with contaminated water or soil. However, the specific transmission pathways in the studied individuals cannot be determined from the present data and remain speculative.

It is also important to highlight that three marmosets (*Callithrix jacchus*) (3/9–33.3%) showed PCR amplification of *Leptospira* spp. DNA in different organs. Among the tissues evaluated, reproductive organs (uterus, testis, and ovary) were positive, with one positive organ detected per individual. However, all PCR-positive marmosets also showed leptospiral DNA in multiple systemic organs, including kidneys, bladder, liver, lung, and heart, indicating generalized dissemination of the pathogen.

Detection of *Leptospira* spp. DNA in reproductive tissues has been reported in other animal species, including livestock and wildlife (24, 25). However, these studies involve different host species, ecological contexts, and diagnostic approaches, which limits direct comparison with the present findings.

Therefore, in the present study, PCR positivity in reproductive organs is more likely to reflect systemic infection rather than specific colonization of the reproductive tract. PCR-based detection alone is insufficient to infer venereal transmission in marmosets, and confirmation of such a route would require additional evidence, such as isolation of viable organisms or detection in reproductive secretions.

Capybaras (*Hydrochoerus hydrochaeris*) are recognized reservoirs of *Leptospira* spp., and it is well established that a large proportion of free-ranging individuals are infected (26). The only capybara evaluated in this study was not reactive in the MAT; however, molecular characterization revealed the presence of the *lipL32* target in the heart and kidneys of this animal. As observed in other cities across Brazil (27), capybaras can also be seen circulating within the urban environment of João Pessoa, capital of the state of Paraíba, particularly near preserved fragments of the Atlantic Forest and along the main river that crosses the city, the Rio Jaguaribe, where low-income populations often occupy irregular settlements along riverbanks (28). Although only one capybara was included in this study, the serological finding is consistent with previous reports of exposure to *Leptospira* spp. in this species. Therefore, this result should be interpreted as supporting previous evidence rather than demonstrating the epidemiological role of capybaras in the study area.

This study has several limitations that should be considered when interpreting the results. First, the sample size for some wildlife species was small, which limits the ability to draw robust species-specific epidemiological conclusions. Second, the sampling design was opportunistic, based on animals admitted to rehabilitation centers, which may introduce selection bias and may not accurately represent free-ranging populations. In addition, the study lacked longitudinal follow-up, preventing the assessment of infection dynamics over time, including persistence or clearance of *Leptospira* spp. infection. The absence of bacterial isolation and genotyping also limits further characterization of circulating strains and confirmation of viable organisms.

Furthermore, there was limited overlap between serological and molecular datasets, which restricts direct comparison between exposure (serology) and active infection (PCR). Finally, the study design does not allow assessment of bacterial shedding, transmission dynamics, or reservoir competence in the evaluated species. Therefore, all interpretations related to epidemiological roles, including potential reservoir status, should be made with caution. Although species-specific prevalence estimates were calculated, the small sample size available for several species resulted in wide confidence intervals, reducing the precision of these estimates. Therefore, these findings should be interpreted with caution and confirmed in future studies with larger sample sizes.

## 5 Conclusion

This study provides serological and molecular evidence of exposure to and infection by pathogenic *Leptospira* spp. in rescued wild mammals from Paraíba, Brazil, demonstrating the circulation of the pathogen among different wildlife species admitted to rehabilitation centers. The detection of leptospiral DNA in multiple tissues, including reproductive organs, reinforces the importance of integrating serological and molecular approaches in wildlife surveillance programs. Although the present study does not directly evaluate ecological or anthropogenic drivers, the findings highlight the potential relevance of wildlife rehabilitation centers as epidemiological interfaces at the human–animal–environment interface from a One Health perspective. These results also emphasize the importance of continuous wildlife surveillance, public health monitoring, and biosafety measures in rehabilitation facilities to reduce potential risks associated with zoonotic pathogen circulation.

Future studies should include larger sample sizes, longitudinal monitoring, bacterial isolation, molecular characterization, and investigations addressing bacterial shedding, transmission dynamics, and the possible epidemiological role of wildlife species in the maintenance of pathogenic *Leptospira* spp.

## Data Availability

The original contributions presented in the study are included in the article/supplementary material, further inquiries can be directed to the corresponding author.
